# Design Solutions for Slender Bars with Variable Cross-Sections to Increase the Critical Buckling Force

**DOI:** 10.3390/ma15176094

**Published:** 2022-09-02

**Authors:** Marius Florin Botis, Camelia Cerbu

**Affiliations:** 1 Department of Civil Engineering, Faculty of Civil Engineering, Transilvania University of Brasov, B-dul Eroilor, No. 29, 500036 Brasov, Romania; 2 Department of Mechanical Engineering, Faculty of Mechanical Engineering, Transilvania University of Brasov, No. 29, B-dul Eroilor, 500036 Brasov, Romania

**Keywords:** buckling, stability, civil engineering, slender bars, columns, numerical analysis, variable cross-section

## Abstract

In large metal civil constructions (stadium roofs, bridges), slender bars can lose their stability under compression loading. There is a lack in the literature regarding design solutions and methods for increasing the critical buckling force of bars with variable cross-sections. The aim of this research is to present a numerical model with finite elements used for a comparative analysis of increasing the critical force of stability loss in cases of (i) bars with stepwise variation in the cross-sections and (ii) bars with continuous variation in the moment of inertia along the bar axis (parabolic, sinusoidal, triangular, and trapezoidal variation). Considering the large-scale applications in civil engineering, bars that were pin-connected at one end and simple-supported at the other end were analyzed. Firstly, the analytical model was described to compute the critical buckling force for bars with stepwise variation in the cross-sections. Then, a finite element model for a slender bar and the assumptions considered were presented. The results were computed using the MATLAB program based on the numerical model proposed and were validated with the analytical model for stepwise variable cross-sections of the bars. The numerical model was adapted for bars with continuous variation in the moment of inertia along the bar axis. It was shown that, by trapezoidal variation in the second moment of inertia along the axis of a bar, i.e., as buckling occurred in the elastic field, the critical buckling force could be increased by 3.556 times compared to a bar with a constant section. It was shown that there was certain bar with stepwise variation in the cross-section for which the critical buckling force was approximately equal to the one obtained for the bar with sinusoidal variation in the moment of inertia (increased by 3.427 times compared to a bar with a constant section).

## 1. Introduction

The loss in stability of elements designed for engineering structures remains of great importance and topicality for various applications in civil engineering (stadium roofs, bridges), shipbuilding, and aerospace construction (aircraft). These items are usually made of steel or alloys with high strength characteristics. Depending on the type of the element (slender column, beam, plate, or shell) and on the type of loading, there are different analytical models and numerical models used for the analysis of their stability.

The first problems regarding elastic instability were approached and solved by L. Euler [1,2] in the middle of the 18th century, over 200 years ago. Nowadays, the main problems regarding the theory of elastic stability for different types of elements (columns, beams, frames, rings, curved bars, arches, thin plates, and thin shells), were synthesized by S. P. Timoshenko and J. M. Gere in a reference book in the scientific literature [2]. The loss in stability of structural elements takes place under the action of compressive loads [3,4] or bending (lateral buckling of beams) [2,5,6]. The optimized designs of such elements involve modifying geometry in order to increase the critical buckling force and ensure a low weight. In the case of steel structures, just decreasing the volume can lead to weight reduction. In the context of the research approached in this article, the control of the variation in the cross-section along the axis of a slender bar subjected to compression is of great importance for increasing the critical buckling force and, last but not the least, for reducing the weight. The problem of designing the shapes of bars concerning the cross-section variation also becomes stringent for bars subjected to compression in tensegrity structures [7] used for roofs in modern civil buildings.

The questions are as follows: which geometric parameter (dimensions, area, or the second moment of inertia) of a cross-section should be considered, and which mathematical function models the continuous variation in the section along the axis of a bar to significantly increase the critical buckling force while keeping the length of the bar and boundary conditions unchanged? What is the best design solution for the shape of a bar: stepwise or continuous variation in the cross-section along the bar axis? Another issue raised is how much the ratio between the critical buckling force and volume of a bar is affected considering the variation in the cross-section along the axis of the bar.

In the last years, many researchers around the world have investigated the stability loss in columns having different boundary conditions and non-uniform cross-sections under the action of axially distributed force [3,4,8]. S. P. Timoshenko and J. M. Gere were among the first researchers who presented a theoretical approach of buckling for bars with changes in the cross-sections without considering an axial distribution of compressive force in the second edition of their book [2] (the first edition of this book was published in 1961).

Eisenberger M. [3] found an exact solution for the buckling loads of columns with polynomial variation in the bending stiffness of the cross-sections under an axial load with a polynomial distribution along the bar axis by considering the determinant of the stiffness matrix to be equal to zero at the stability loss. Considering the Euler–Bernoulli beam theory to model a column with a variable moment of inertia I(x) (linear or parabolic variation) in the rectangular cross-section along its axis, which is subjected to distributed axial force, Darbandi et al. [8] computed buckling loads, taking into account the Wentzel–Kramers–Brillouin method of singular perturbation. Just the buckling loads and corresponding mode shapes for the rectangular variable cross-sections of columns were reported in that research, and those results were compared with the results given by Eisenberger [3].

Coskun and Atay [9] used a variational integration method to compute the normalized critical buckling load for Euler’s columns with variable cross-sections with different boundary conditions, considering that the flexural stiffness varied by exponential function or by power function (linear, quadratic, and cubic variation). Their results matched very well with the exact solutions, but the paper did not customize the solutions for different shapes of cross-sections (for example, rectangular, circular, and annular cross-sections). Regarding computation methods, Ma et al. [10] computed critical buckling force considering various higher-order shear deformation beam theories based on Engesser’s hypothesis and Haringx’s hypothesis in comparison with Euler’s theory in order to show the effects of warping shape. That research showed that buckling loads were not influenced by the theory used in the case of very slender columns or in the case of those having high shear rigidity.

Taking into account the achievements of the research mentioned above, we focus on finding an analysis method that allows the accurate calculation of the critical buckling force both for a bar with a stepwise variable cross-section and for a bar with a continuous variable cross-section. The analysis model must be flexible in terms of easy adaptation for any function used for variation in the second moment of inertia of the cross-section along the axis. This is the reason why the research focuses on the use of a finite element method for the numerical analysis of a slender bar subjected to compression in order to compute the critical buckling force. However, only a program made with specific software for calculation (such as MATLAB) can ensure the necessary flexibility, not a commercial one used for finite element analysis. Another problem arises concerning the validation method. In this context, it is necessary to present the state-of-the-art methods in the literature regarding the numerical methods used to analyze the stability of columns subjected to compression.

Using a finite difference method, Soltani and Sistani [11] also investigated a stability analysis for columns having variable flexural stiffnesses subjected to variable axial force. In that research, a finite difference method was applied in the case of column having a rectangular cross-section or an I-shaped cross-section whose dimensions were variable along the axis of the column in order to compute the critical buckling load. On the other hand, in nanomechanics, there are specific methods to analyze the buckling of nanobeams resting on elastic substrate media [12].

Saraçaoğlu and Uzun [13] showed critical buckling loads obtained with Ansys 19.0 software for certain columns having square or circular cross-sections that were variable along the axis of the column (linear variation combined with a portion having a constant cross-section). Szmidla et al. [14] investigated and showed results concerning a stability analysis for steel columns consisting of portions having inhomogeneous cross-sections (composed cross-sections). Regarding columns with inhomogeneous cross-sections, Li et al. [15] made a parametric optimization of composite columns against buckling.

For columns consisting of certain portions having constant cross-sections, Maalawi [16] approached the optimization of buckling calculation in order to obtain design variables (area of the cross-section, length of each portion, radius of gyration) for the maximization of the critical buckling load and for certain input data (number of portions, boundary conditions, cross-section type).

A localized differential quadrature method was used by Yilmaz et al. [17] in order to compute non-dimensional critical buckling loads for non-uniform columns with continuous elastic restraint and different boundary conditions.

Using a discretized Hencky bar-chain model and a parallel genetic algorithm, Ruocco et al. [4] provided in 2017 an optimization method against buckling for columns with non-uniform variation in the cross-section subjected to both distributed and concentrated compressive forces. In this case, the geometrical variation in the cross-section was not given as input data. The paper [4] reported the optimal variation in the circular cross-section in non-dimensional coordinates and its corresponding non-dimensional normalized buckling load by keeping the same length and volume of the column with a uniform cross-section.

In the literature [18], optimization methods were also presented for columns having thin-walled, open cross-sections in order to obtain the maximum critical buckling by considering the constraint that the volume of the column remained constant.

As mentioned before, a retrospective analysis of the literature highlights the fact that there is a lack of research on design methods and solutions to increase the critical buckling force of slender bars with annular cross-sections subjected to compression by continuous variation in the dimensions of the cross-sections along the axis of the bars. Furthermore, no comparative studies have been reported regarding the critical buckling force for slender bars with continuous annular cross-sections compared to bars with stepwise variable cross-sections.

The present research aims to make some contributions to the scientific literature regarding design solutions for slender bars with variable cross-sections along the bar axis. The research provides a numerical model with finite elements validated by an analytical model, which is used to compute the critical buckling forces both for bars with stepwise variation in the cross-section and for bars with continuous variation in the cross-section along the bar axis. The MATLAB program used in this research based on the finite element model presented allows easy adaptation for any variation function considered for the moment of inertia. Considering variable annular cross-sections, the MATLAB program calculates additionally the inner and outer diameters that define the geometry of the bar. Moreover, a comparison is shown of the critical buckling forces for different particular cases considered for variation in the cross-section. In this way, the comparative analysis leads to finding some functions for variation in the second moment of inertia of the cross-sections, which ensure a major increase in the critical buckling force.

In this context, the main purpose of the research is to present a numerical model with finite elements used for a comparative analysis of increasing the critical buckling force for two types of slender bars subjected to compression: (i) bars with stepwise variation in the cross-section and (ii) bars having annular cross-sections with continuous variation in the moment of inertia along the bar axis. The results are also compared with the values of the critical buckling forces corresponding to bars having constant cross-sections along the bar axis. The case of bars that are pin-connected at one end and simple-supported at the other end is taken into account, considering the wide range of applications of these kinds of bars in engineering construction (stadium roofs, bridges, aircraft, and so on).

The main objectives of the research are: (i) the generalization of an analytical model for the calculation of the critical buckling force for a slender bar with stepwise variation in the cross-section, as well as a bar consisting of three portions; (ii) a numerical analysis using the MATLAB program, which uses modeling with finite elements for a slender bar with a stepwise variable cross-section along the axis of the bar in order to obtain the critical buckling force; (iii) the validation of the finite element model (FEM), with the results obtained with an analytical model for a bar with stepwise variation in the cross-section; (iv) an adaptation of the MATLAB program for a numerical analysis of a bar with a continuous variable cross-section; and (v) a comparison of the results regarding the critical buckling force for slender bars with stepwise variation and with continuous variation in the cross-sections. It is considered that the dimensions of a cross-section vary along the bar axis as the second moment of inertia I(x) varies from I to 4I. For a slender bar with continuous variation in the cross-section, the following types of variation are considered for the second moment of inertia I(x) along the bar axis: parabolic, sinusoidal, triangular, and trapezoidal. The numerical simulation with FEM is made using a computer program made with MATLAB R2014a software.

All the results are interpreted in terms of the normalized critical buckling force with respect to the critical force for stability loss corresponding to the bars having constant cross-sections along the axes of the bars. It is assumed that all the bars involved in this study lose their stability in the elastic field.

Finally, this research reports accurate results on increasing the normalized critical buckling force for slender bars with stepwise or continuous variations in the cross-sections involved in this study. In this way, this research shows design solutions regarding the mathematical functions for variation in the second moment of inertia of a cross-section along the bar axis in order to increase the critical buckling force for slender bars subjected to compressive loads.

## 2. Work Methods

### 2.1. Analytical Model for Buckling of a Column with Pin Connections at Ends with Stepwise Variable Section

**Figure 1:**
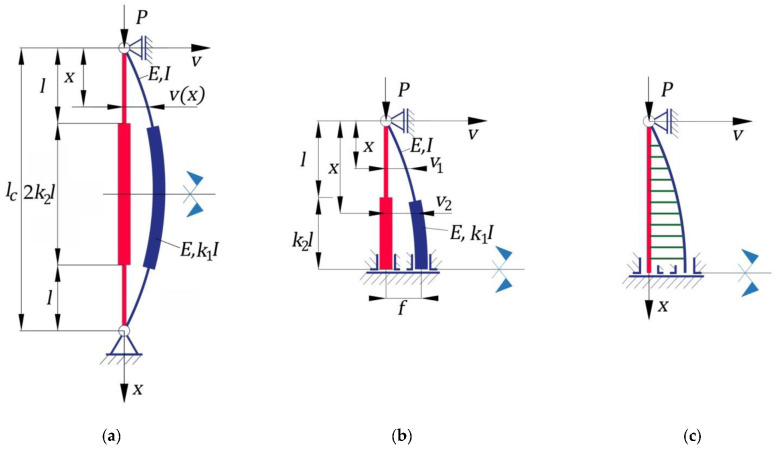
Bar analyzed having a stepwise variable cross-section whose bottom end is pin-connected, while the upper end is simply supported: (**a**) geometrical model of the entire bar and deformed shape at stability loss; (**b**) geometrical model considering the symmetry condition; (**c**) bending-moment diagram.

In Figure 1, a geometrical model is shown of a bar having a stepwise variable circular cross-section whose bottom end is pin-connected, while the upper end is simply supported. The undeformed and deformed shapes of the bar under compression are shown in Figure 1a. The bar consists in three portions. The first and the third portions have the same values for length l and for the second moment of inertia I of the cross-section. The second bar portion has a cross-section whose moment of inertia $I_{1}$ is equal to $k_{1}I$, with $k_{1}>1$, and its length is equal to $2k_{2}l$. In other words, the parameters $k_{1}$ and $k_{2}$ represent the ratios of the lengths and of the second moment of inertia, respectively, corresponding to the second and first portions of the bar (Figure 1a). The total length of the column is denoted with $l_{c}$ (Figure 1a). All portions of the bar are made of the same isotropic material having a modulus of elasticity E. The column is symmetric with respect to its midpoint (Figure 1a), and consequently, the analysis model may be reduced to half of the bar, considering the symmetry conditions (Figure 1b). The shape of the bending-moment diagram for half of the bar is shown in Figure 1c.

The main purpose of this subsection is to compare the critical buckling force corresponding to a column having a stepwise variable circular cross-section with the critical buckling force corresponding to a column having the same length and a constant cross-section whose moment of inertia is equal to I. For this purpose, the main objectives are to compute the critical buckling force corresponding to the column having a stepwise variable cross-section and to compare it with the one corresponding to the column with a constant cross-section, as well as an analysis of the rational shapes for buckling in the case of columns having stepwise variable circular cross-sections.

Due to symmetry, the buckling analysis was made by considering a model of half of a column (Figure 1b) with boundary conditions corresponding to the midpoint of the column located on the horizontal symmetry axis.

It was assumed that the column buckled in the elastic domain. This meant that Bernoulli’s hypothesis and Euler’s relation were valid at buckling.

The bending moment $M_{b1}\left(x\right)$ developed at buckling and caused by the compressive force *P* at the level of the arbitrary cross-section located on the first portion of the column was computed using Equation (1):

(1) $$M_{b1}\left(x\right)=-Pv_{1}\left(x\right),$$

where $v_{1}\left(x\right)$ represents the deflection function of the arbitrary cross-section of the first column portion located at distance x with respect to the end of the column (Figure 1b).

In the same manner, the bending moment $M_{b2}\left(x\right)$ developed at buckling at the level of the arbitrary cross-section located on the second portion of the column was computed using Equation (2):

(2) $$M_{b2}\left(x\right)=-Pv_{2}\left(x\right),$$

where $v_{2}\left(x\right)$ represents the deflection function of the arbitrary cross-section of the second column portion located at distance x with respect to the end of the column (Figure 1b).

To compute the critical buckling force, we used an analytical method. The differential equation of the approximate deformed median fiber corresponding to the first portion of the bar is given in Equation (3):

(3) $$\frac{d^{2}v_{1}\left(x\right)}{dx^{2}}=-\frac{M_{b1}\left(x\right)}{EI}.$$

Replacing Equation (1), Equation (3) became the following [2]:

(4) $$\frac{d^{2}v_{1}\left(x\right)}{dx^{2}}+\frac{Pv_{1}\left(x\right)}{EI}=0.$$

In the same manner, the differential equation of the approximate deformed median fiber corresponding to the second portion of the bar is given in Equation (5) [2]:

(5) $$\;\frac{d^{2}v_{2}\left(x\right)}{dx^{2}}+\frac{Pv_{2}\left(x\right)}{k_{1}EI}=0.$$

The notation α was introduced for the ratio given in Equation (6) [2]:

(6) $$\alpha =\sqrt{\frac{P}{k_{1}EI}}\;\;\;\;\;\mathrm{or}\;\;\;\;\;\alpha^{2}=\frac{P}{k_{1}EI}.$$

By using Equation (6), differential Equations (4) and (5) of the approximate deformed median fibers for the bar portions became:

1. Equation (7) for the first portion of the column [2]:
  (7) $$\frac{d^{2}v_{1}\left(x\right)}{dx^{2}}+{\left(\alpha \sqrt{k_{1}}\right)}^{2}\cdot v_{1}\left(x\right)=0,$$
2. Equation (8) for the second portion of the column [2]:
  (8) $$\frac{d^{2}v_{2}\left(x\right)}{dx^{2}}+\alpha^{2}\cdot v_{2}\left(x\right)=0.$$

Solutions of inhomogeneous second-order differential Equations (7) and (8) were given by Equations (9) and (10) for the first portion and for the second portion of the column, respectively:

(9) $$v_{1}\left(x\right)=C_{1}\sin\left(\alpha \sqrt{k_{1}}x\right)+C_{2}\cos\left(\alpha \sqrt{k_{1}}x\right),$$

(10) $$v_{2}\left(x\right)=C_{3}\sin\left(\alpha x\right)+C_{4}\cos\left(\alpha x\right).$$

Integration constants $C_{i}\;\left(i=\bar{1,\;4}\right)$ were computed using the boundary conditions given in Equation (11):

(11) $$\{\begin{array}{ll} \mathrm{for}\;x=0, & v_{1}\left(0\right)=0;\\ \mathrm{for}\;x=l+k_{2}l, & v_{2}\left(l+k_{2}l\right)=f;\\ \mathrm{for}\;x=l+k_{2}l, & \frac{dv_{2}}{dx}\left(l+k_{2}l\right)=0, \end{array}$$

where f represents the deflection of the midpoint of the bar at buckling. The continuity conditions for the deformed shape of the median fiber of the column are given in Equation (12) at the level of the bar cross-section located at distance l with respect to the upper simply supported end of the column:

(12) $$\{\begin{array}{ll} \mathrm{for}\;x=l, & v_{1}\left(l\right)=v_{2}\left(l\right);\\ \mathrm{for}\;x=l, & \frac{dv_{1}}{dx}\left(l\right)=\frac{dv_{2}}{dx}\left(l\right). \end{array}$$

The first derivatives of the functions $v_{1}\left(x\right)$ and $v_{2}\left(x\right)$ of the deflections of the arbitrary cross-section corresponding to each column portion were computed using Equations (9) and (10), respectively. In fact, these derivatives represented the functions of the rotations of the arbitrary cross-section and were expressed by Equations (13) and (14):

(13) $$\frac{dv_{1}\left(x\right)}{dx}=\alpha \sqrt{k_{1}}C_{1}\left[\cos\left(\alpha \sqrt{k_{1}}x\right)-C_{2}\sin\left(\alpha \sqrt{k_{1}}x\right)\right],$$

(14) $$\frac{dv_{2}\left(x\right)}{dx}=\alpha \left[C_{3}\cos\left(kx\right)-C_{4}\sin\left(\alpha x\right)\right].$$

By replacing Equations (9), (10), (13), and (14) in the boundary conditions given by Equation (11) and in the continuity conditions given in Equation (12), the system of Equation (15) was obtained:

(15) $$\{\begin{array}{l} \begin{array}{l} C_{2}=0;\\ C_{3}\sin\left[\alpha l\left(1+k_{2}\right)\right]+C_{4}\cos\left[\alpha l\left(1+k_{2}\right)\right]=f; \end{array}\\ \alpha \left\{C_{3}\cos\left[\alpha l\left(1+k_{2}\right)\right]-C_{4}\sin\left[\alpha l\left(1+k_{2}\right)\right]\right\}=0;\\ \begin{array}{l} C_{1}\sin\left(\alpha l\sqrt{k_{1}}\right)+C_{2}\cos\left(\alpha l\sqrt{k_{1}}\right)=C_{3}\sin\left(\alpha l\right)+C_{4}\cos\left(\alpha l\right);\\ \alpha \sqrt{k_{1}}\left[C_{1}\cos\left(\alpha l\sqrt{k_{1}}\right)-C_{2}\sin\left(\alpha l\sqrt{k_{1}}\right)\right]=\alpha \left[C_{3}\cos\left(\alpha l\right)-C_{4}\sin\left(\alpha l\right)\right], \end{array} \end{array}$$

whose unknown quantities are the integration constants $C_{i}\;\left(i=\bar{1,\;4}\right)$ and the deflection *f* of the midpoint of the column.

Equation system (15) was reduced practically to the following system of four equations:

(16) $$\{\begin{array}{l} \begin{array}{l} C_{3}\sin\left[\alpha l\left(1+k_{2}\right)\right]+C_{4}\cos\left[\alpha l\left(1+k_{2}\right)\right]-f=0;\\ C_{3}\cos\left[\alpha l\left(1+k_{2}\right)\right]-C_{4}\sin\left[\alpha l\left(1+k_{2}\right)\right]=0; \end{array}\\ \begin{array}{l} C_{1}\sin\left(\alpha l\sqrt{k_{1}}\right)-C_{3}\sin\left(\alpha l\right)-C_{4}\cos\left(\alpha l\right)=0;\\ C_{1}\sqrt{k_{1}}\cos\left(\alpha l\sqrt{k_{1}}\right)-C_{3}\cos\left(\alpha l\right)+C_{4}\sin\left(\alpha l\right)=0. \end{array} \end{array}$$

The homogeneous system of Equation (16) had nonzero solutions for $C_{i}\;\left(i=\bar{1,\;4}\right)$ and *f* if the determinant of the coefficients was zero. This condition led to Equation (17):

(17) $$\mathrm{Det}\;\left[\begin{array}{cccc} 0 & \sin\left[\alpha l\left(1+k_{2}\right)\right] & \cos\left[\alpha l\left(1+k_{2}\right)\right] & -1\\ 0 & \cos\left[\alpha l\left(1+k_{2}\right)\right] & -\sin\left[\alpha l\left(1+k_{2}\right)\right] & 0\\ \sin\left(\alpha l\sqrt{k_{1}}\right) & -\sin\left(\alpha l\right) & -\cos\left(\alpha l\right) & 0\\ \sqrt{k_{1}}\cos\left(\alpha l\sqrt{k_{1}}\right) & -\cos\left(\alpha l\right) & \sin\left(\alpha l\right) & 0 \end{array}\right]=0.$$

The notation ξ was introduced and computed with Equation (18):

(18) ξ=αl,

and Equation (17) became:

(19) $$\mathrm{Det}\left[\begin{array}{cccc} 0 & \sin\left[\xi \left(1+k_{2}\right)\right] & \cos\left[\xi \left(1+k_{2}\right)\right] & -1\\ 0 & \cos\left[\xi \left(1+k_{2}\right)\right] & -\sin\left[\xi \left(1+k_{2}\right)\right] & 0\\ \sin\left(\xi \sqrt{k_{1}}\right) & -\sin\xi & -\cos\xi & 0\\ \sqrt{k_{1}}\cos\left(\xi \sqrt{k_{1}}\right) & -\cos\xi & \sin\xi & 0 \end{array}\right]=0,$$

whose unknown is *ξ*. To compute the critical buckling force, the minimum value of the absolute values of the solutions *ξ* must be used because, for other solutions *ξ*, the value of the critical force would be greater.

$\xi_{min}$ denoted the minimum value of the absolute values of the solutions *ξ* of Equation (19).

From Equation (18), the minimum value of α could be computed using Equation (20):

(20) $$\alpha_{min}=\xi_{\min}/l.$$

Equation (20) was replaced in relation (6) in order to compute the critical buckling force for a column having a stepwise variable cross-section:

(21) $$P_{cr}=k_{1}\alpha_{\min}^{2}EI=k_{1}\xi_{\min}^{2}\frac{EI}{l^{2}}.$$

The length l of the first portion was expressed in the function of the total length $l_{c}$ of the column using Equation (22) according to Figure 1a:

(22) $$l=l_{c}/\left[2\left(1+k_{2}\right)\right].$$

Equation (22) was replaced in Equation (21) in order to compute the critical buckling force $P_{cr}$ for a column having a stepwise variable cross-section:

(23) $$P_{cr}=4k_{1}{\left(1+k_{2}\right)}^{2}\xi_{\min}^{2}\frac{EI}{l_{c}^{2}}={\left(\frac{2\sqrt{k_{1}}\left(1+k_{2}\right)\xi_{\min}^{}}{\pi }\right)}^{2}\frac{\pi^{2}EI}{l_{c}^{2}}.$$

On the other hand, the critical buckling force $P_{cr0}$ for a column with a constant cross-section whose moment of inertia of the section was I having the same total length $l_{c}$ with pin connections at its ends was computed as follows:

(24) $$P_{cr0}=\pi^{2}EI/l_{c}^{2}.$$

Using Equations (23) and (24), the normalized buckling force (denoted with *c*) was computed as being equal with the ratio between the critical buckling force $P_{cr}$ for a column having a stepwise variable cross-section and the critical buckling force $P_{cr0}$ for a column having a constant cross-section whose moment of inertia of the section was I:

(25) $$c=\frac{P_{cr}}{P_{cr0}}={\left(\frac{2\sqrt{k_{1}}\left(1+k_{2}\right)\xi_{\min}^{}}{\pi }\right)}^{2}.$$

It is said that a column is rationally designed if the critical buckling force is increased while the mass of the material of the column is optimal. In structure design, both the mass of the material, which influences the material costs, and the weight of the structure are also important. The manufacturing costs increase due to the additional manufacturing operations required for a column having a stepwise variable cross-section.

In this research, it was said that a column having a stepwise variable cross-section was rationally designed if its ratio between the critical buckling force $P_{cr}$ and the total volume V of the column was greater than the similar ratio computed for a column with a constant cross-section whose moment of inertia of the section was I with the same total length $l_{c}$.

The ratio between the critical buckling force $P_{cr}$ and the total volume V of the column was computed as follows, taking into account the geometry of the column shown in Figure 1a:

(26) $$\frac{P_{cr}}{V}=\frac{P_{cr}}{2l\left(A+A_{1}k_{2}\right)},$$

where A represents the cross-sectional areas for the first and the third portions of the column having the length l, while $A_{1}$ represents the area of the cross-section for the second column portion whose length is equal to $2k_{2}l$.

It was assumed that each portion of the column had a circular cross-section. It may be remarked in Equation (27), which gives the ratio between the moment of inertia I and the second power of the area of the cross-section A for the first portion, whose diameter is denoted with d:

(27) $$\frac{I}{A^{2}}=\frac{\pi d^{4}/64}{{\left(\pi d^{2}/4\right)}^{2}}=\frac{1}{4\pi }$$

or

(28) $$I=A^{2}/4\pi .$$

In the same manner, Equation (29) was written for the second portion of the column:

(29) $$I_{1}=A_{1}^{2}/4\pi .$$

On the other hand, the relation between the moments of inertia *I*_1_ and *I* corresponding to the first two portions of the column, respectively, is given in Equation (30):

(30) $$I_{1}=k_{1}I.$$

Equations (28) and (29) were replaced in Equation (30), and it obtained Equation (31):

(31) $$A_{1}=A\sqrt{k_{1}}.$$

Then, relations (22) and (31) were replaced in relation (26), which became:

(32) $$\frac{P_{cr}}{V}=\frac{\left(1+k_{2}\right)P_{cr}}{l_{c}A\left(1+k_{2}\sqrt{k_{1}}\right)}.$$

From Equation (25), the critical buckling force $P_{cr}$ for a column having a stepwise variable cross-section could be computed in the function of the critical buckling force $P_{cr0}$ of a column with a constant cross-section:

(33) $$P_{cr}=cP_{cr0}.$$

The volume of the column with a constant cross-section was:

(34) $$V_{0}=l_{c}A.$$

Replacing Equations (33) and (34) in Equation (32) obtained Equation (35):

(35) $$\frac{P_{cr}}{V}=\frac{\left(1+k_{2}\right)c}{1+k_{2}\sqrt{k_{1}}}\frac{P_{cr0}}{V_{0}},$$

which led to Equation (36), which computed the rationality factor $k_{rat}$:

(36) $$k_{rat}=\frac{P_{cr}/V}{P_{cr0}/V_{0}}=\frac{\left(1+k_{2}\right)c}{1+k_{2}\sqrt{k_{1}}}\;.$$

Replacing the ratio *c* given by Equation (25) in Equation (36) led to Equation (37):

(37) $$k_{rat}=\frac{P_{cr}/V}{P_{cr0}/V_{0}}=\frac{\left(1+k_{2}\right)}{1+k_{2}\sqrt{k_{1}}}{\left(\frac{2\sqrt{k_{1}}\left(1+k_{2}\right)\xi_{\min}^{}}{\pi }\right)}^{2}=\frac{4\xi_{\min}^{2}k_{1}{\left(1+k_{2}\right)}^{3}}{\pi^{2}\left(1+k_{2}\sqrt{k_{1}}\right)},$$

which was used to compute the ratio between the rationality factor $P_{cr}/V$ corresponding to a column having a stepwise variable cross-section and the rationality factor $P_{cr0}/V_{0}$ corresponding to a column with a constant cross-section.

### 2.2. Numerical Modeling and Simulation for Loss in Stability of a Column with Pin Connections at Ends with Stepwise Variable Cross-Section

If the compressive stress, which acts on the slenderness of a column, is variable, then the relation between force and displacement may be written with Equation (38):

(38) $$\left\{dP\right\}=\left[K_{T}\right]\left\{∆U\right\},$$

where infinitesimal variation in the force is denoted with dP. A similar relation may be written with Equation (39):

(39) $$\left\{∆P\right\}=\left[K_{s}\right]\left\{∆U\right\},$$

where finite variation in the force is denoted with ∆P. In Equations (38) and (39), $\left[K_{T}\right]=\left[K_{T}\left\{U\right\}\right]$ and $\left[K_{T}\right]=\left[K_{T}\left\{U\right\}\right]$ represent the tangent stiffness matrix and the secant stiffness matrix, respectively. The finite variation in the displacements was computed with Equation (40):

(40) $$\left\{∆U\right\}={\left[K_{T}\left(U\right)\right]}^{-1}\left\{∆P\right\}=\frac{{\left[K_{T}\left(U\right)\right]}^{*}}{\det\left[K_{T}\left(U\right)\right]}\left\{∆P\right\},$$

where ${\left[K_{T}\left(U\right)\right]}^{*}$ is the adjunct matrix of the stiffness matrix.

The phenomenon of loss in stability (transition from one equilibrium shape to another equilibrium shape) takes place when the displacements tend toward infinity for a variation ∆P in the compressive force. From a mathematical point of view, this condition is fulfilled if the determinant of the tangent stiffness matrix $\left[K_{T}\right]$ is equal to zero, which was expressed by Equation (41):

(41) $$\det\left[K_{T}\left(U\right)\right]=0.$$

Equation (41) could be written with Equation (42):

(42) $$\det\left[\left[K\right]-\lambda \left[K_{g}\right]\right]=0,$$

where [K] represents the elastic stiffness matrix of the structure (e.g., the column) obtained by assembling of the stiffness matrices $\left[K_{e}\right]$ corresponding to the finite elements that form the structure; $\left[K_{g}\right]$ is the geometric stiffness matrix of the structure obtained by assembling of the geometric stiffness matrices $\left[K_{ge}\right]$ corresponding to the finite elements that form the structure; and λ is the common multiplier of the axial forces *N* acting in the slender bar.

**Figure 2:**
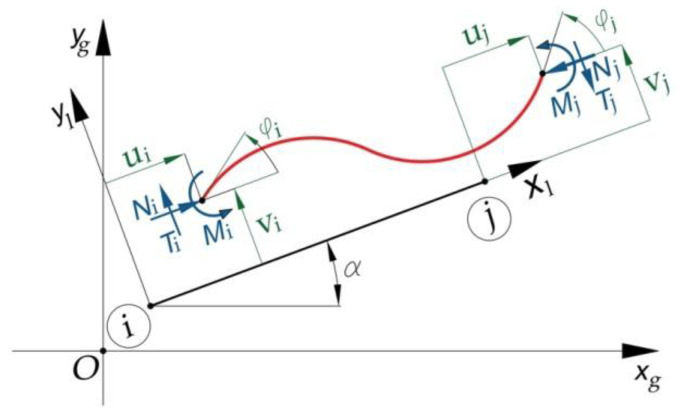
Characteristics of the finite elements of the bar double-embedded at both ends in terms of both the internal forces and flections (displacement and rotation) developed at the nodes.

For the finite element of the double-embedded bar type (Figure 2), the elastic stiffness matrix $\left[K_{e}\right]$ and the geometric stiffness matrix $\left[K_{ge}\right]$ were expressed with Equations (43) and (44), respectively:

(43) $$\left[K_{ge}\right]=\left[\begin{array}{cc} \begin{array}{ccc} \frac{{EA}^{\prime }}{l^{\prime }} & 0 & 0\\ 0 & \frac{12EI^{\prime }}{{l^{\prime }}^{3}} & \frac{6EI^{\prime }}{{l^{\prime }}^{2}}\\ 0 & \frac{6EI^{\prime }}{{l^{\prime }}^{2}} & \frac{4EI^{\prime }}{l^{\prime }} \end{array} & \begin{array}{ccc} -\frac{{EA}^{\prime }}{l^{\prime }} & 0 & 0\\ 0 & -\frac{12EI^{\prime }}{{l^{\prime }}^{3}} & \frac{6EI^{\prime }}{{l^{\prime }}^{2}}\\ 0 & -\frac{6EI^{\prime }}{{l^{\prime }}^{2}} & \frac{2EI^{\prime }}{l^{\prime }} \end{array}\\ \begin{array}{ccc} -\frac{{EA}^{\prime }}{l^{\prime }} & 0 & 0\\ 0 & -\frac{12EI^{\prime }}{{l^{\prime }}^{3}} & -\frac{6EI^{\prime }}{{l^{\prime }}^{2}}\\ 0 & \frac{6EI^{\prime }}{{l^{\prime }}^{2}} & \frac{2EI^{\prime }}{l^{\prime }} \end{array} & \begin{array}{ccc} \frac{EA^{\prime }}{l^{\prime }} & 0 & 0\\ 0 & \frac{12EI^{\prime }}{l^{\prime 3}} & -\frac{6EI^{\prime }}{l^{\prime 2}}\\ 0 & -\frac{6EI^{\prime }}{l^{\prime 2}} & \frac{4EI^{\prime }}{l^{\prime }} \end{array} \end{array}\right]$$

(44) $$\left[K_{ge}\right]=\frac{N}{l}\left[\begin{array}{cc} \begin{array}{ccc} 0 & 0 & 0\\ 0 & \frac{6}{5} & \frac{l^{\prime }}{10}\\ 0 & \frac{l^{\prime }}{10} & \frac{2{l^{\prime }}^{2}}{15} \end{array} & \begin{array}{ccc} 0 & 0 & 0\\ 0 & -\frac{6}{5} & \frac{l^{\prime }}{10}\\ 0 & -\frac{l^{\prime }}{10} & -\frac{{l^{\prime }}^{2}}{30} \end{array}\\ \begin{array}{ccc} 0 & 0 & 0\\ 0 & -\frac{6}{5} & -\frac{l^{\prime }}{10}\\ 0 & \frac{{\mathrm{ll}}^{\prime }}{10} & -\frac{{l^{\prime }}^{2}}{30} \end{array} & \begin{array}{ccc} 0 & 0 & 0\\ 0 & \frac{6}{5} & -\frac{{\mathrm{ll}}^{\prime }}{10}\\ 0 & -\frac{{\mathrm{ll}}^{\prime }}{10} & \frac{2{l^{\prime }}^{2}}{15} \end{array} \end{array}\right]$$

where $l^{\prime }$, $A^{\prime }$, and $I^{\prime }$ are the length, area of the cross-section, and the second moment of inertia of the cross-section, respectively, corresponding to the finite elements double-embedded at both ends.

In this context, the solving of the stability equation involved solving a problem of eigenvectors and eigenvalues. The solutions of the stability equation were the eigenvalues $\lambda_{k}\;\left(k=\bar{1,\;n}\right)$ corresponding to the multiplier of the axial forces. For the eigenvalues $\lambda_{k}\;\left(k=\bar{1,\;n}\right)$, the corresponding eigenvectors $\left\{U_{k}\right\}\;\left(k=\bar{1,\;n}\right)$ were determined, which represented the geometric shapes (equilibrium shapes) of the loss in stability. From a practical point of view, only the lowest eigenvalue $\lambda_{\min}$ was of interest, the other values being of interest just from a theoretical point of view.

The numerical model for the calculation of the critical buckling force was validated for a bar having a pin connection at one end and a simple support at the other end. Considering the numerical model previously described, a computer calculation program was written with MATLAB R2014a software for the calculation of the eigenvalues and eigenvectors for the loss in stability of a slender bar subjected to compression. Because just the first value of the critical buckling force and the corresponding deformed shape were of interest, the calculation program reported just the first three values of the critical buckling force and plotted the corresponding eigenvectors.

The assumptions considered in the numerical analysis of the finite elements were the following: (i) the material of the bar was isotropic, homogeneous, and linearly elastic; (ii) the hypothesis of small strains was valid; and (iii) the normal stress $\sigma_{p}$ at the proportionality limit was approximately equal to the normal stress at yielding, denoted with $f_{y}$, for the material of the bar.

In order to obtain the numerical solution with the finite element method, the main steps covered by the MATLAB program were the following: (i) meshing of the bar in a certain number of finite elements; (ii) computing both the elastic stiffness matrix $\left[K_{e}\right]$ and the geometric stiffness matrix $\left[K_{ge}\right]$ corresponding to each finite element according to Equations (43) and (44), respectively; (iii) assembling all the stiffness matrices $\left[K_{e}\right]$ and geometric stiffness matrices $\left[K_{ge}\right]$ in order to obtain the elastic stiffness matrix [K] and geometric stiffness matrix $\left[K_{g}\right]$ of the column analyzed; (iv) computing the eigenvalues $\lambda_{k}\;\left(k=\bar{1,\;n}\right)$ by solving Equation (42); (v) computing the eigenvectors $\left\{U_{k}\right\}\left(k=\bar{1,\;n}\right)$ using Equation (40); and (vi) by using the eigenvalues $\lambda_{k}$, computing the critical buckling forces $P_{cr}$. The smallest critical buckling force corresponded to the minimum eigenvalue $\lambda_{\min}$.

In order to validate the numerical model, the loss in stability was analyzed using the numerical model with 18 finite elements for a bar having a pin connection at one end and a simple support at the other end, for which the geometrical characteristics and the material properties are given in Table 1. Considering the geometrical characteristics of the bar given in Table 1, the following quantities were computed: the area A  of the cross-section of 2826 mm^2^; the second moment of inertia *I* of the cross-section, whose value was 2,896,650 mm^4^; and the radius i of inertia, having a value of 32.01562 mm.

For the bar involved, the slenderness ratio λ was computed with Equation (45):

(45) λ=l/i=8000/32.01562=249.878.

For steel of type S355, whose properties are shown in Table 1, the slenderness ratio $\lambda_{0}$ that limited buckling in the elastic field was computed with Equation (46):

(46) $$\lambda_{0}=\pi \sqrt{E/\sigma_{p}}=\pi \sqrt{E/f_{y}}=76.37041,$$

where it is assumed that the normal stress $\sigma_{p}$ at the proportionality limit is approximately equal to the normal stress at yielding, denoted with $f_{y}$, for the material of the bar (Table 1).

**Figure 3:**
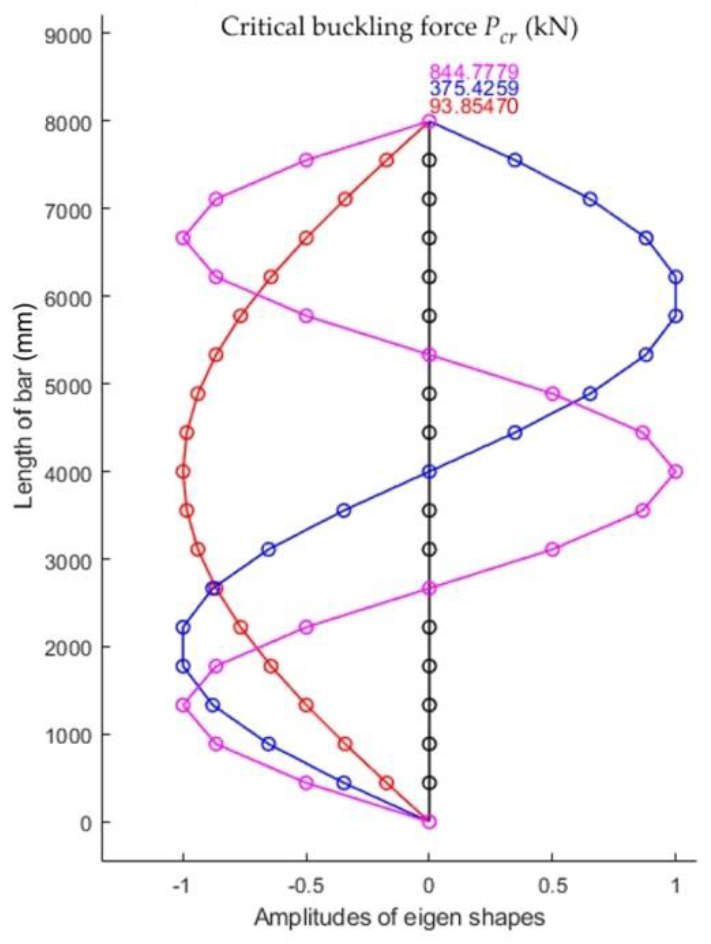
The first three shapes of stability loss and corresponding critical buckling forces for the bar, whose geometrical and material characteristics are given in Table 1.

The first three eigenshapes and the corresponding eigenvalues for the bar analyzed are shown in Figure 3.

**Figure 4:**
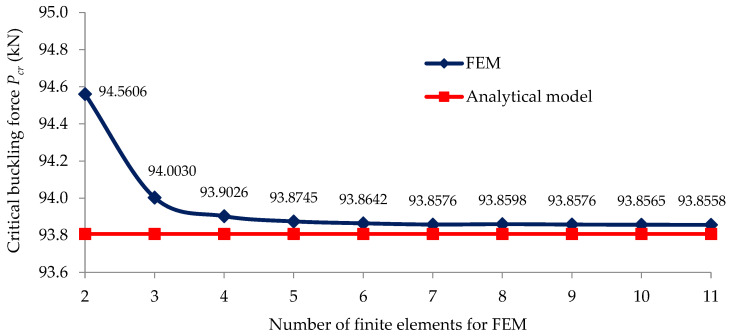
Analysis concerning the convergence of the solution obtained for the critical buckling load by the FEM compared with respect to the value computed with the analytical model.

In Figure 4, the convergence of the critical buckling load $P_{cr}$ obtained with the algorithm of the numerical model is shown related to the number of the finite elements of the numerical model, and it is analyzed with respect to the critical buckling load of 93.807 kN computed with Equation (24) using the analytical model. By analyzing Figure 4, it can be remarked that the solutions obtained through numerical modeling with the FEM tended asymptotically to the value of 93.86 kN for the numerical model, which had at least six elements. It can be concluded that the numerical model consisting of 18 finite elements provided results that were sufficiently accurate concerning the critical buckling load.

Six cases of bars with pin connections at one end and simple supports at the other end, shown in Table 2, were analyzed using the numerical model with finite elements in order to show the effects of a stepwise variable cross-section on the critical force of stability loss.

In Table 2, two extreme cases were considered for bars whose the second moments of inertia were constant along the bar length l: (i) a bar having a second moment of inertia equal to I (code CONST_1I); and (ii) a bar having a second moment of inertia equal to 4I (code CONST_1I). For both cases, the length l of the bar was that given in Table 1, and it was assumed that the bar had an annular cross-section. The value I for the second moment of inertia corresponded to an annular cross-section having an inner diameter d and an outer diameter D, which are also given in Table 1. For the second bar, for which the second moment of inertia was equal to 4I, the inner diameter d and outer diameter D were computed by considering the area A of the annular cross-section in order for the normal stress in compression to be equal to the normal stress $\sigma_{p}$ at the proportionality limit (which was approximately equal to the normal stress at yielding $f_{y}$).

The results obtained using the numerical model with 32 finite elements were comparatively analyzed for all the types of columns shown in Table 2.

### 2.3. Numerical Modeling and Simulation for Loss in Stability of a Column with Pin Connections at Ends with Continuous Variable Cross-Section

#### 2.3.1. Cases Approached and Assumptions concerning the Variation in the Second Moment of Inertia

Four cases of variation in the second moment of inertia I(x) were investigated: (i) parabolic variation; (ii) sinusoidal variation; (iii) triangular variation; (iv) trapezoidal variation; and (v) constant variation.

It was assumed that the arbitrary cross-section of a bar whose area was *A*(*x*) was an annular cross-section having an inner diameter *d*(*x*) and an outer diameter *D*(*x*). The length of the bar was denoted with *l*. Assuming that the bar must lose its stability in the elastic field (for which Euler’s relation is valid), the normal stress *σ* developed at the arbitrary point of the cross-section must be smaller or equal to the normal stress at the proportionality limit denoted with *σ_p_*, which was considered to be equal to the normal stress at yielding $f_{y}$ (given in Table 1 for S355 steel). In this context, the area A(x) of the cross-section was computed with Equation (47), considering that the normal stress *σ* developed at that cross-section was equal to the normal stress $\sigma_{p}$ at the proportionality limit for the material of the bar at stability loss:

(47) $$A\left(x\right)=P_{cr}/\sigma_{p},$$

where $P_{cr}$ is the critical buckling force (or the critical force of stability loss).

For the arbitrary cross-section of the bar, knowing the area A(x) computed with Equation (47) and the variation in the second moment of inertia I(x), the inner and outer diameters of the cross-section were computed with Equations (48) and (49), respectively [20]:

(48) $$d\left(x\right)=\sqrt{\frac{8I\left(x\right)}{A\left(x\right)}-\frac{2A\left(x\right)}{\pi }},$$

(49) $$D\left(x\right)=\sqrt{\frac{8I\left(x\right)}{A\left(x\right)}+\frac{2A\left(x\right)}{\pi }}.$$

Equations (48) and (49) show that the wall thickness of the bar cross-section changed along the axis of the bar as long as the ratio between d(x) and D(x) was not constant.

To analyze the critical buckling force $P_{cr}$ for a column with pin connections at both ends whose second moment of inertia I had continuous variation along the axis of the bar, numerical models were used that were similar to the one corresponding with the bar with stepwise variation in the cross-section. In order to analyze the solution convergence for the critical buckling force $P_{cr}$, the numerical analysis was repeated for FEMs consisting of 20, 80, 100, 200, 400, and 800 elements, respectively. The convergence analysis made for FEMs corresponding to bars with continuous variation in the second moment of inertia along the bar axis led to the conclusion that the solutions for the critical buckling force converged for FEMs consisting of a minimum of 100 elements.

#### 2.3.2. Parabolic Variation in the Second Moment of Inertia of the Cross-Section along the Bar

The consideration of the second moment of inertia I(x) of an arbitrary cross-section with parabolic variation along the bar axis is given by Equation (50):

(50) $$I\left(x\right)=Ax^{2}+Bx+C,$$

where x represents the position of the arbitrary cross-section with respect to the pin-connected end of the bar.

The constants A, B, C were computed considering that the second moment of inertia was equal with I for the cross-sections of the bar ends, and it was equal to 4I for the middle of the bar. These conditions are given by Equation (51):

(51) $$\{\begin{array}{ll} \mathrm{for}\;x=0, & I\left(0\right)=C=I;\\ \mathrm{for}\;x=l/2, & I\left(l/2\right)=A{\left(l/2\right)}^{2}+Bl/2+C=4I;\\ \mathrm{for}\;x=l, & I\left(l\right)=Al^{2}+Bl+C=I, \end{array}$$

and led to the constants given by Equation (52):

(52) $$\begin{array}{ccc} A=-12I/l^{2}; & B=12I/l; & C=I \end{array}.$$

**Figure 5:**
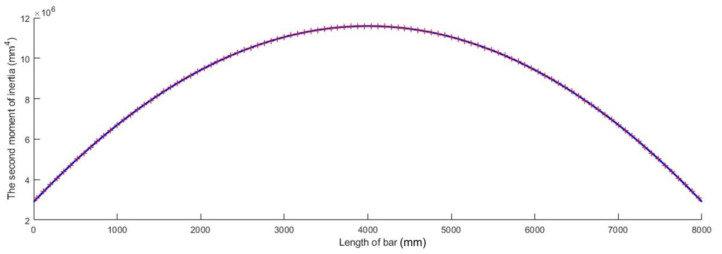
Parabolic variation in the second moment of inertia *I* along the bar axis.

By replacing the above constants A, B, and C in Equation (50), the parabolic variation in the second moment I(x) along the axis of the bar is given by Equation (53):

(53) $$I\left(x\right)=-\left(12I/l^{2}\right)x^{2}+\left(12I/l\right)x+I,$$

which is plotted in Figure 5 for a bar whose length l is given in Table 1 and whose second moment of inertia I of the cross-sections located at the bar ends was computed for an annular cross-section having an inner diameter d and an outer diameter D, which are also given in Table 1.

**Figure 6:**
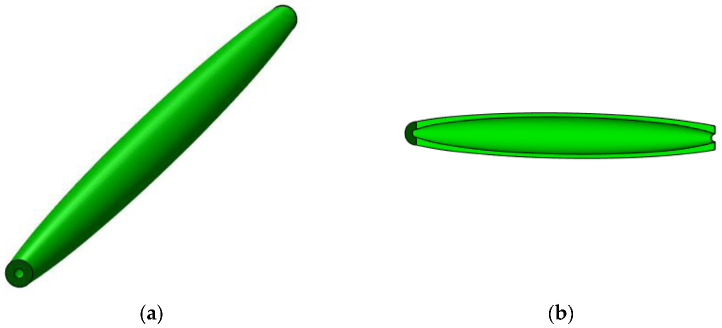
Geometrical model for a bar with parabolic variation in the second moment of inertia along the bar axis: (**a**) isometric view and (**b**) longitudinal section.

In Figure 6, a geometrical model is shown of a bar whose parabolic variation in the second moment of inertia I(x) is given by Equation (53).

#### 2.3.3. Sinusoidal Variation in the Second Moment of Inertia of the Cross-Section along the Bar

The variation in the second moment of inertia I(x) of an arbitrary cross-section along the bar axis as a sinusoidal function is given by Equation (54):

(54) I(x)=3Isin(πx/l)+I.

Considering the conditions given by Equation (55), it could be checked that the second moment of inertia was equal to I for the cross-sections located at the bar ends, and it was 4I for the cross-section located at the middle of the bar:

(55) $$\{\begin{array}{ll} \mathrm{for}\;x=0; & I\left(0\right)=3I\sin\left(\pi \cdot 0/l\right)+I=I;\\ \mathrm{for}\;x=l/2; & I\left(l/2\right)=3I\sin\left(\pi \left(l/2\right)/l\right)+I=3I\sin\left(\pi /2\right)+I=4I;\\ \mathrm{for}\;x=l; & I\left(l\right)=3I\sin\left(\pi l/l\right)+I=3I\sin\pi +I=I. \end{array}$$

**Figure 7:**
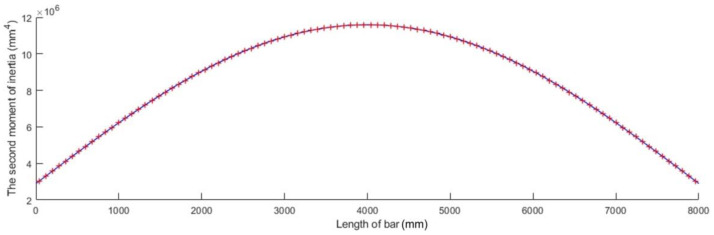
Sinusoidal variation in the second moment of inertia along the bar axis.

The sinusoidal variation in the second moment I(x) along the axis of the bar given by Equation (54) is plotted in Figure 7 for a bar whose length l is given in Table 1 and whose second moment of inertia I of the cross-sections located at bar ends was computed for an annular cross-section having an inner diameter d and an outer diameter D, which are also given in Table 1.

**Figure 8:**
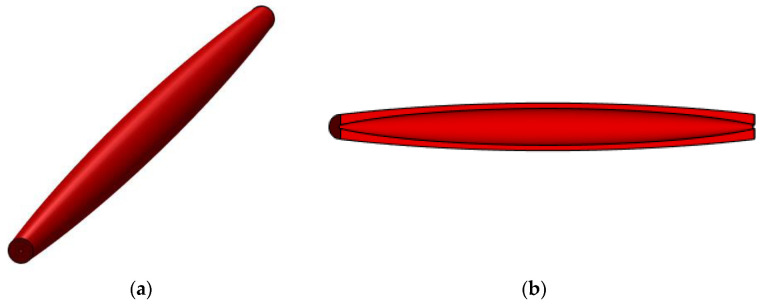
Geometrical model for s bar with sinusoidal variation in the second moment of inertia along the bar axis: (**a**) isometric view and (**b**) longitudinal section.

In Figure 8, a geometrical model is shown of a bar whose sinusoidal variation in the second moment of inertia I(x) is given by Equation (54).

#### 2.3.4. Triangular Variation in the Second Moment of Inertia of the Cross-Section along the Bar

It was considered that the second moment of inertia linearly increased from the value I to the value 4I from the pin-connected end of the bar to the middle of the bar, which meant position *x* of the cross-section was in the range of [0,l/2]. Then, the second moment of inertia linearly decreased from the value 4I to the value I from the middle of the bar to the other end of the bar, which meant a variation of x in the range of [l/2, l].

A linear function for the second moment of inertia for the first half of the bar, which meant x∈(0, l/2), and it was given by Equation (56):

(56) $$I_{1}\left(x\right)=Ax+B,$$

whose constants A and B are computed using the conditions given by Equation (57) regarding the values of the second moments of inertia for the cross-sections located at the pin-end connection and at the middle of the bar:

(57) $$\{\begin{array}{ll} \mathrm{for}\;x=0; & I_{1}\left(0\right)=A\cdot 0+B=I;\\ \mathrm{for}\;x=l/2; & I_{1}\left(l/2\right)=Al/2+B=4I. \end{array}$$

Using the conditions given by Equation (57), the constants A and B were computed, and the results are given in Equation (58):

(58) $$\begin{array}{cc} A=6I/l; & B=I. \end{array}$$

By replacing the constants A and B in Equation (56), a linear function was obtained for the second moment of inertia for the first half of the bar, which is given in Equation (59):

(59) $$\begin{array}{cc} I_{1}\left(x\right)=6Ix/l+I, & \mathrm{for}\;x\in \left(0,\;0.5l\right). \end{array}$$

A linear function was assumed for the second moment of inertia for the second half of the bar, which meant x∈(0.5,l), and it was given by Equation (60):

(60) $$I_{2}\left(x\right)=Cx+D,$$

whose constants C and D are computed by using the conditions given by Equation (61) regarding the values of the second moments of inertia for the cross-sections located at the middle of the bar and at bar end, which is simple-supported:

(61) $$\{\begin{array}{ll} \mathrm{for}\;x=l/2; & I_{2}\left(l/2\right)=Cl/2+D=4I;\\ \mathrm{for}\;x=l; & I_{2}\left(l\right)=Cl+D=I. \end{array}$$

Using the conditions given by Equation (61), the constants C and D were computed, and the results are given in Equation (62):

(62) $$\begin{array}{cc} C=-6I/l; & D=7I. \end{array}$$

By replacing the constants C and D in Equation (60), a linear function was obtained for the second moment of inertia for the first half of the bar, which is given in Equation (63):

(63) $$\begin{array}{cc} I_{2}\left(x\right)=-6Ix/l+7I, & \mathrm{for}\;x\in \left(0.5l,\;l\right) \end{array}.$$

**Figure 9:**
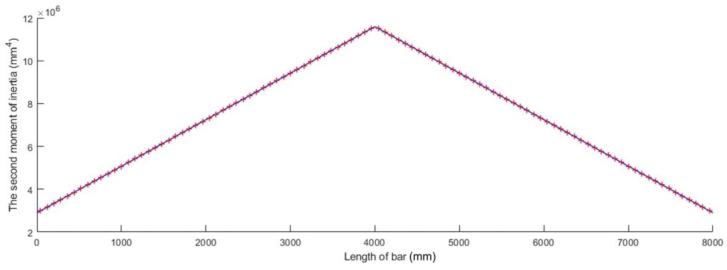
Triangular variation in the second moment of inertia along the bar axis.

The triangular variation in the second moment I(x) along the axis of the bar given by Equations (59) and (63) is plotted in Figure 9 for a bar whose length l is given in Table 1 and whose second moment of inertia I of the cross-sections located at the bar ends was computed for an annular cross-section having an inner diameter d and an outer diameter D, which are also given in Table 1.

**Figure 10:**
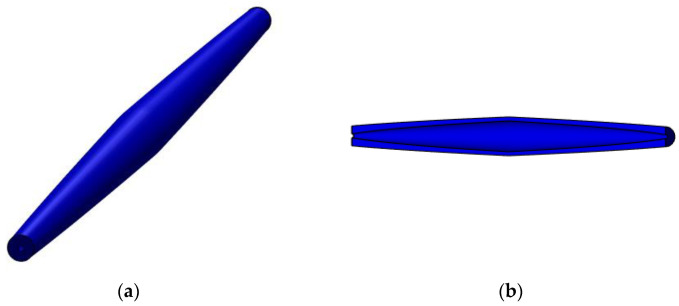
Geometrical model for a bar with triangular variation in the second moment of inertia along the bar axis: (**a**) isometric view and (**b**) longitudinal section.

In Figure 10, a geometrical model is shown of a bar whose triangular variation in the second moment of inertia I(x) is given by Equations (59) and (63).

#### 2.3.5. Trapezoidal Variation in the Second Moment of Inertia of the Cross-Section along the Bar

For trapezoidal variation in the second moment of inertia I(x) along the axis of a bar, three portions of the bar length l were considered in order to obtain the functions for such a variation: (i) for the first third of the bar, which meant xϵ(0,l/3), the second moment of inertia linearly increased from the value I to the value 4I; (ii) for the second third of the bar, which meant xϵ(l/3, 2l/3), the cross-section remained constant, and the second moment of inertia was equal with 4I; and (iii) for the last portion, which meant xϵ(2l/3, l), the second moment of inertia linearly decreased from the value 4I to the value I.

The linear function corresponding to the first portion is given in Equation (64):

(64) $$I_{1}\left(x\right)=Ax+B,$$

for which the constants A and B are computed using the conditions written in Equation (65):

(65) $$\{\begin{array}{ll} \mathrm{for}\;x=0; & I_{1}\left(0\right)=A\cdot 0+B=I;\\ \mathrm{for}\;x=l/3; & I_{1}\left(l/3\right)=Al/3+B=4I. \end{array}$$

Solving the above system of two equations led to the following values for the constants A and B:

(66) $$\begin{array}{cc} A=9I/l; & B=I. \end{array}$$

By replacing the constants A and B in Equation (64), a linear function was obtained of the second moment of inertia corresponding to the first portion of the bar, as shown in Equation (67):

(67) $$\begin{array}{cc} I_{1}\left(x\right)=9Ix/l+I, & \mathrm{for}\;x\in \left[0,l/3\right] \end{array}.$$

The function for the second moment of inertia corresponding to the second portion of the bar is given by Equation (68):

(68) $$\begin{array}{cc} I_{2}\left(x\right)=4I, & \mathrm{for}\;x\;\in \;\left[l/3,\;2l/3\right] \end{array}.$$

For the third portion of the bar, the function of the second moment of inertia was assumed, as given by Equation (69):

(69) $$I_{3}\left(x\right)=Cx+D,$$

for which constants C and D are computed using the following conditions:

(70) $$\{\begin{array}{ll} \mathrm{for}\;x=2l/3; & I_{3}\left(l/3\right)=2Cl/3+D=4I;\\ \mathrm{for}\;x=l; & I_{3}\left(l\right)=Cl+D=I. \end{array}$$

By solving the system of two equations given by Equation (70) and replacing constants C and D in Equation (69), the following function of the second moment of inertia was obtained:

(71) $$\begin{array}{cc} I_{3}\left(x\right)=-9Ix/l+10I, & \mathrm{for}\;x\in \left[2l/3,l\right] \end{array}.$$

**Figure 11:**
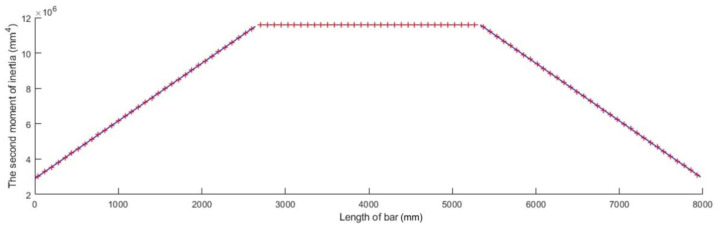
Trapezoidal variation in the second moment of inertia along the bar axis.

**Figure 12:**
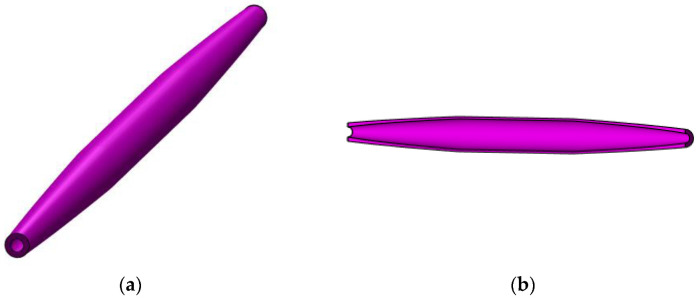
Geometrical model for a bar with trapezoidal variation in the second moment of inertia along the bar axis: (**a**) isometric view and (**b**) longitudinal section.

Considering the functions given by Equations (67), (68), and (71), trapezoidal variation in the second moment of inertia along the bar axis is graphically shown in Figure 11 for a bar whose length l is given in Table 1 and whose second moment of inertia I of the cross-sections located at the bar ends was computed for an annular cross-section having an inner diameter d and an outer diameter D, which are also given in Table 1. The geometrical model of such a bar is shown in Figure 12.

## 3. Results

### 3.1. Results Obtained by Analytical Model

**Figure 13:**
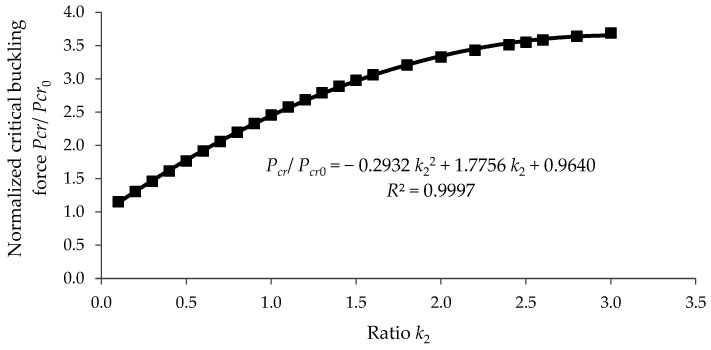
Variation in the normalized critical buckling force $P_{cr}/P_{cr0}$. computed with Equation (25) considering $k_{1}=4$ related to the ratio $k_{2}$.

Figure 13 shows the variation in the ratio $P_{cr}/P_{cr0}$. computed with Equation (25) for the case of $k_{1}=4$. related to the value $k_{2}\in \left[0;3\right]$. It may be observed that, for this design of a column with a variable cross-section, the maximum value of the ratio $P_{cr}/P_{cr0}$. was equal to 3.69 in the case when $k_{2}$. was equal to 3.

**Figure 14:**
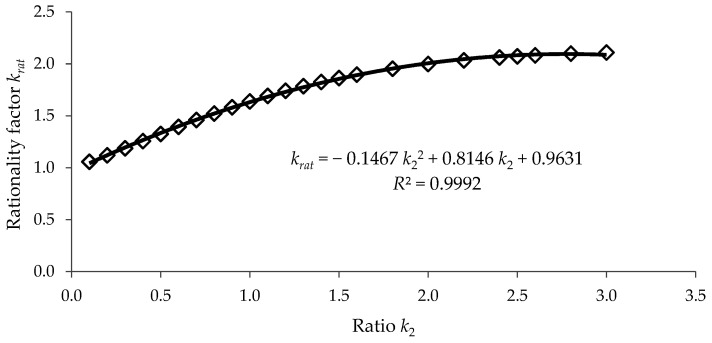
Variation in the rationality factor $k_{rat}$. computed with Equation (37) considering $k_{1}=4$. related to the ratio $k_{2}$.

Figure 14 shows the variation in the rationality factor $k_{rat}$ computed with Equation (37) for the case of $k_{1}=4$. related to the value $k_{2}\in \left[0;3\right]$. It was observed that the column with a stepwise variable cross-section was more rationally designed because the rationality factor $k_{rat}$. was always greater than 1, and the greatest value was 2.1086 in the case when $k_{2}$. was equal to 3. In fact, for the ratio $k_{2}$. in the range of [2, 3], the rationality factor $k_{rat}$. varied between 1.9973 and 2.1086.

The least squares method was used for the approximation of the data in both Figure 13 and Figure 14 considering the second-degree polynomial functions. The approximation functions both for the normalized critical buckling load $P_{cr}/P_{cr0}$ and for the rationality factor $k_{rat}$. are given in Figure 13 and Figure 14, where the value *R*^2^ close to 1 shows that the data were accurately approximated.

### 3.2. Results by Numerical Modeling for Loss in Stability of a Bar with Pin Connections at Ends with Stepwise Variable Cross-Section

**Figure 15:**
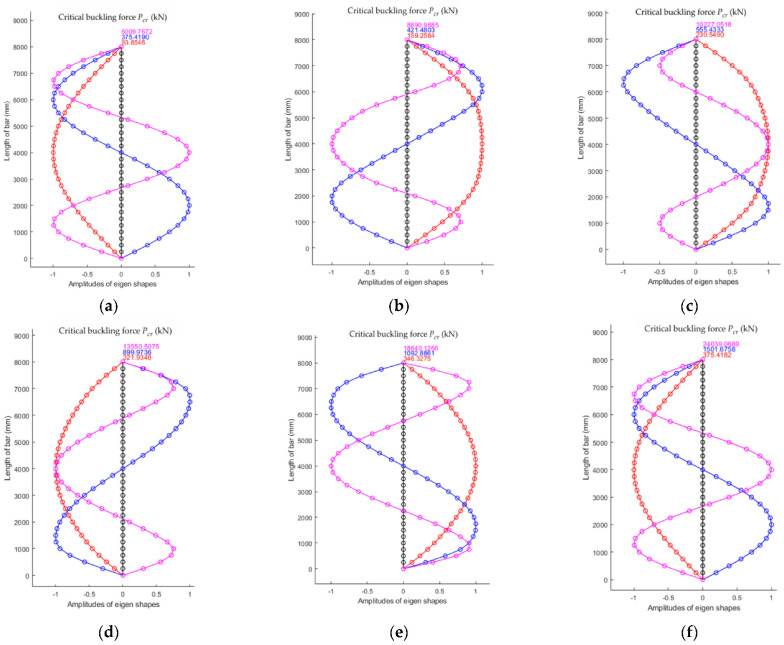
The first three shapes of stability loss and corresponding critical forces $P_{cr}$. obtained by the FEM for the six cases of bars analyzed: (**a**) CONST_1I; (**b**) STEPWISE405; (**c**) STEPWISE410; (**d**) STEPWISE420; (**e**) STEPWISE430; and (**f**) CONST_4I (details about each case are given in Table 2).

Considering the column with pin connections at its ends and a stepwise variable cross-section shown in Figure 1, the results obtained using the MATLAB calculation program for the numerical model with 32 finite elements are plotted in Figure 15 for all six cases given in Table 2.

### 3.3. Validation of the Numerical Model by Theoretical Results for a Column with Stepwise Variable Cross-Section

Table 3 shows the results obtained for the critical buckling force $P_{cr}$. using both the FEM and the analytical model in the cases of columns with stepwise variable cross-sections and with constant cross-sections involved in this research. It was observed that the numerical model was validated by the results obtained with the analytical model because the maximum error was equal to 3.84%, as shown in Table 3.

**Figure 16:**
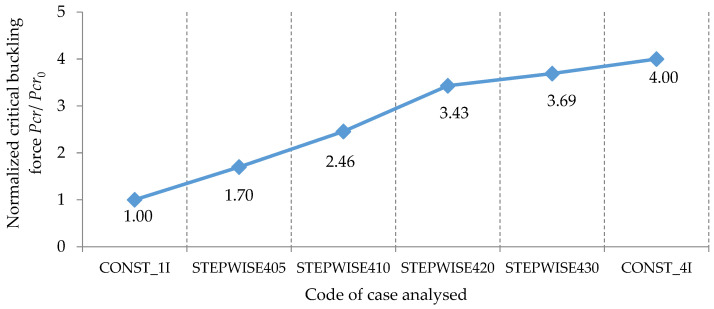
Comparison of the normalized critical buckling forces $P_{cr}/P_{cr0}$. computed with the finite element models for the bars involved (details about each case are given in Table 2).

**Figure 17:**
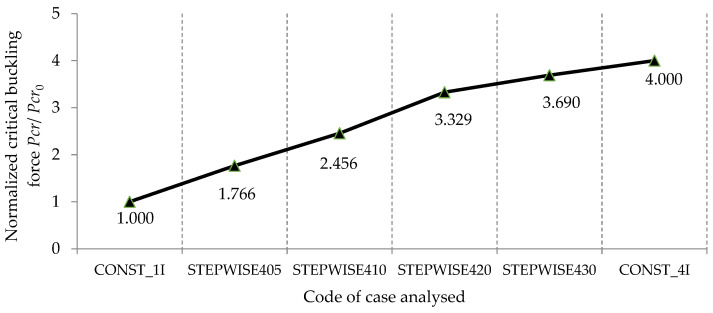
Comparison of the normalized critical buckling forces $P_{cr}/P_{cr0}$ computed with the analytical model for the bars involved in this research (details about each case are given in Table 2).

The values of the normalized critical buckling forces obtained by the FEM and by the analytical model are comparatively plotted in Figure 16 and Figure 17, respectively, for the columns with stepwise variable cross-sections and with constant cross-sections. These graphs also show a good correlation between the numerical model and the analytical model.

### 3.4. Results of Numerical Modeling for Loss in Stability of a Column with Pin Connections at Ends with Continue Variable Cross-Section

#### 3.4.1. Critical Buckling Forces for the Parabolic Variation in the Second Moment of Inertia of the Cross-Section along the Bar

**Figure 18:**
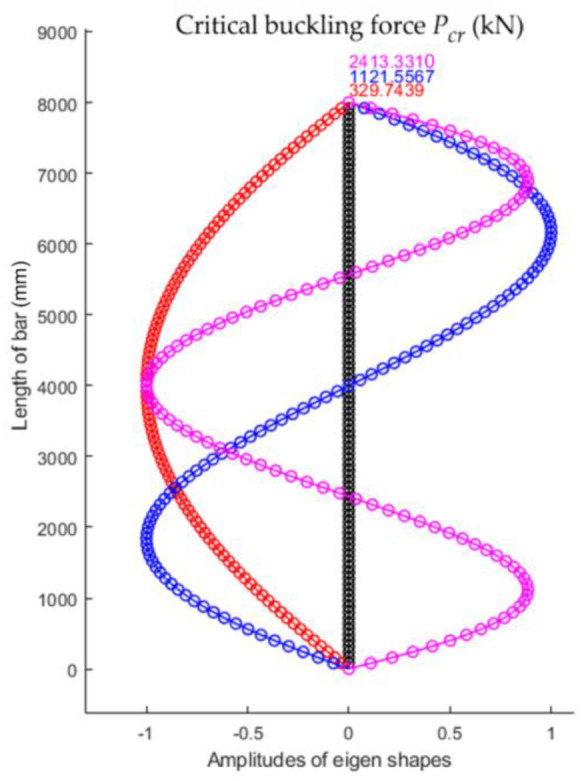
The first three shapes of stability loss and corresponding critical buckling forces for the bar with parabolic variation in the second moment of inertia along the bar axis.

Using the numerical model with 100 finite elements for the bar with parabolic variation in the second moment of inertia, the first three eigenshapes of stability loss were obtained that corresponded to the first three values for the critical buckling force shown in Figure 18. It was observed that the smallest value of the critical force for stability loss $F_{cr}$. was equal to 329.7439 kN.

#### 3.4.2. Critical Buckling Forces for the Sinusoidal Variation in the Second Moment of Inertia of the Cross-Section along the Bar

**Figure 19:**
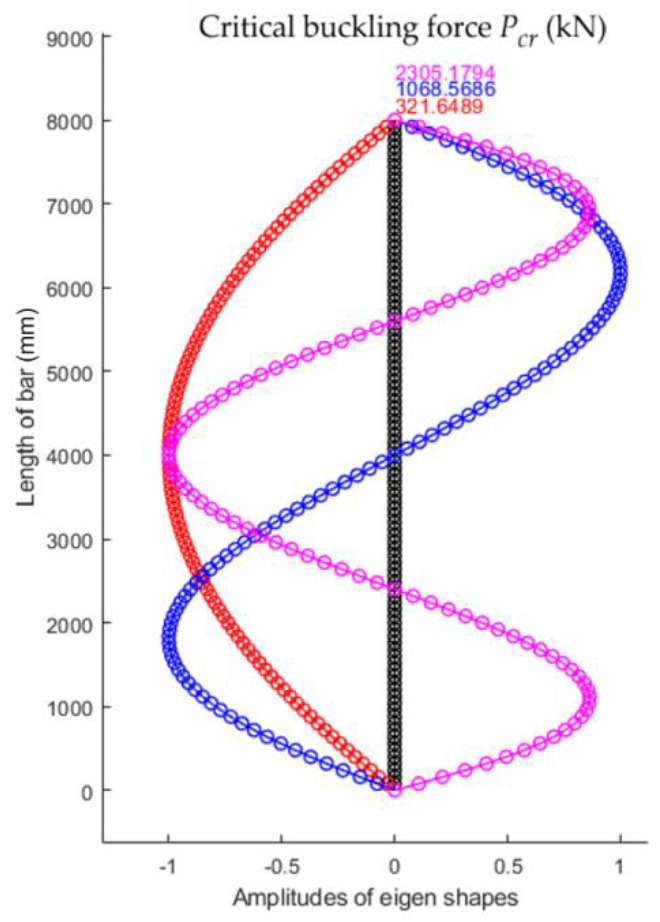
The first three shapes of stability loss and corresponding critical buckling forces for the bar with sinusoidal variation in the second moment of inertia along the bar axis.

Using the numerical model with 100 finite elements, for the bar with sinusoidal variation in the second moment of inertia, the first three eigenshapes of stability loss were obtained that corresponded to the first three values for the critical buckling force shown in Figure 19. It was observed that the smallest value of the critical force for stability loss $F_{cr}$ was equal to 321.6489 kN.

#### 3.4.3. Critical Buckling Forces for the Triangular Variation in the Second Moment of Inertia of the Cross-Section along the Bar

**Figure 20:**
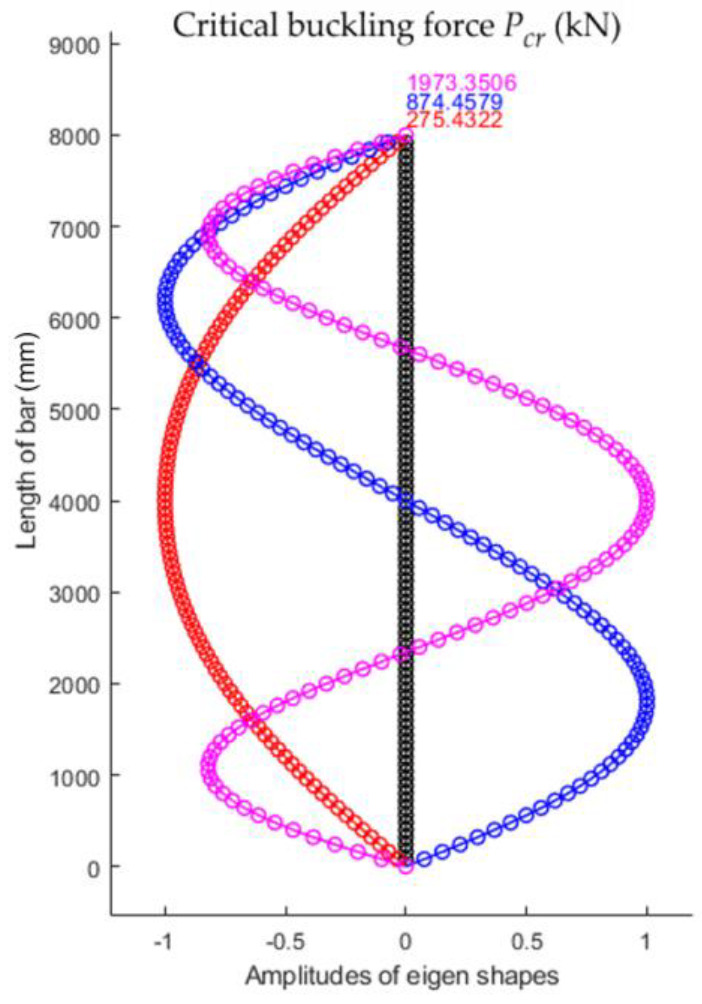
The first three eigenshapes of stability loss and corresponding values for the critical buckling forces for the bar with triangular variation in the second moment of inertia along the bar axis.

Using the numerical model with 100 finite elements for the bar with triangular variation in the second moment of inertia, the first three eigenshapes of stability loss were obtained that corresponded to the first three values for the critical buckling force shown in Figure 20. It was observed that the smallest value of the critical force for stability loss $F_{cr}$. was equal to 275.4322 kN.

#### 3.4.4. Critical Buckling Forces for the Trapezoidal Variation in the Second Moment of Inertia of the Cross-Section along the Bar

**Figure 21:**
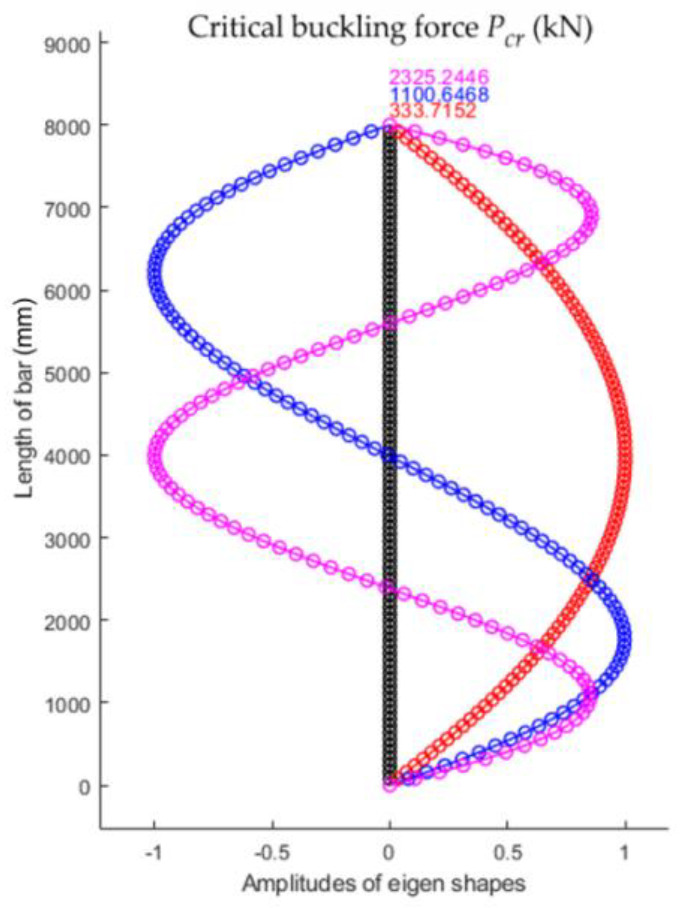
The first three shapes of stability loss and corresponding values for the critical buckling force for the bar with trapezoidal variation in the second moment of inertia along the bar axis.

Using the numerical model with 100 finite elements for the bar with trapezoidal variation in the second moment of inertia, the first three shapes of stability loss corresponding to the first three values of the critical buckling force were obtained and are shown in Figure 21. These were obtained using the numerical model with finite elements of the bar with trapezoidal variation in the second moment of inertia along the bar axis. The smallest value of the critical force for stability loss $F_{cr}$. was equal to 333.7152 kN.

## 4. Discussion

**Figure 22:**
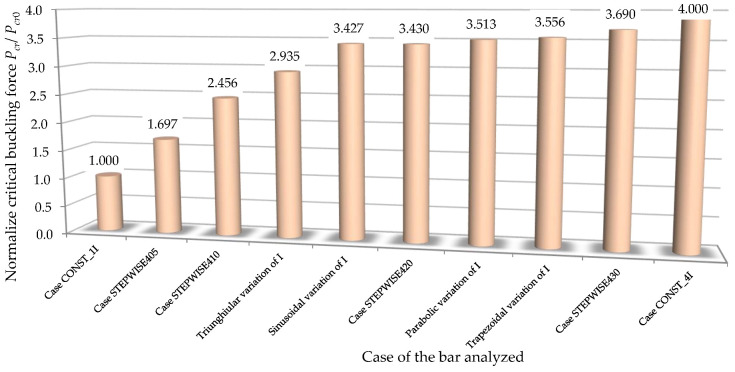
Comparison of the critical buckling force obtained by the FEM for all the cases of bars involved in this research.

In Figure 22, the values of the normalized critical buckling forces $P_{cr}/P_{cr0}$ are comparatively analyzed for all the bars involved in this theoretical research in order to establish the best shapes of both bars with stepwise variable cross-sections and with continuous variable cross-sections, which can lead to a significant increase in the critical buckling force.

By analyzing the results shown in Figure 22, it can be observed that the normalized critical buckling force of 3.43 obtained for the bar with stepwise variation in the cross-section corresponding to case STEPWISE420 was approximately equal to the normalized critical buckling force of 3.427 obtained for the bar with sinusoidal variation in the second moment of inertia along the bar axis from *I* to the maximum value of 4*I*. This remark is very important as long as the bar with the stepwise variable cross-section is more easily obtained from a technological point of view.

Considering Figure 22, it may be also remarked that the normalized critical buckling force of 3.556 recorded for the bar with trapezoidal variation in the second moment of inertia along the bar axis was close to the normalized critical buckling force of 3.513 obtained for the bar with parabolic variation in the second moment of inertia along the bar axis.

For the bars with variable cross-sections involved in this research, the highest value of 3.69 for the normalized critical buckling force was recorded for the bar with stepwise variation in the cross-section corresponding to case STEPWISE430 (Figure 22).

## 5. Conclusions

The research presented in this paper is very important for the design of shapes of slender bars that can lose their stability under compression loads. Increasing the critical buckling force for columns with annular cross-sections by stepwise or continuous variation in the cross-sections along the bar axes was proposed. Moreover, the shapes of the slender bars were designed so that these bars lost their stability in the elastic field.

A particular case was considered for which the second moment of inertia varied between the values I and 4I along the bar axis. For bars with variation in the cross-sections in three steps, the normalized critical buckling force continuously increased by a second-degree polynomial function reported in the paper related to the ratio $k_{2}$ between the lengths of the bar portions. It was shown that the geometry proposed for the bar with stepwise variation in the cross-section was more rational with respect to the bar with a constant cross-section, taking into account the ratio between the critical buckling force and the volume of the bar.

Among the bars involved in this research with continuous variations in the annular cross-sections, it was concluded that the parabolic or trapezoidal variations in the cross-sections were the best solutions. These design solutions led to increases in the critical buckling force by 3.513 times and 3.556 times, respectively, compared with the bar with a constant cross-section along the axis. In fact, the normalized critical buckling forces obtained for both parabolic and trapezoidal variations were close to the one corresponding to case STEPWISE420 with stepwise variation in the cross-section, which was more technologically affordable.

The numerical analysis approach in this research was one of an appropriate design solution to optimize the variation in the cross-section along the axis of a bar by comparing the results in order to maximize the critical buckling force. The numerical model was validated by an analytical model for the bar with stepwise variation in the cross-section, and then the numerical approach was applied for bars with different methods of variation in the second moment of inertia along the bar axis. In fact, the continuous variation in the cross-section was approximated with stepwise variation in the cross-section considering that the bar consisted of a number of portions equal to the number of finite elements considered in the numerical analysis. The main advantage was that the MATLAB program used in this research was not a commercial one, and as a result, the algorithm could be further adapted for other types of variation in the cross-section along the bar axis.

At a time when the rapid printing of construction elements has taken off around the world in the field of construction, the results presented in this paper are of great interest in many applications, taking into account that these results could lead to increasing the critical buckling force for compressed slender bars. However, there is still a need for 3D-printing equipment for construction to be perfected, developed, and made more affordable in terms of cost so that the manufacture of bars with variable cross-sections is easy for construction elements whose shapes have been optimized.

Considering the design solutions for continuous variation in the cross-section presented in this article, it is possible to manufacture sleeves to be welded on compressed bars of various structures (stadium roofs, steel bridge structures, trusses) to increase the critical buckling force. The sleeves may be welded without removing the bars from the structures. Because the research in this article was limited to slender bars with pin connections at both ends, the study may be continued for other boundary conditions in further research.

## Figures and Tables

**Table 1:** Geometrical characteristics and material properties for the bar analyzed by numerical modeling.

A. Geometrical Characteristics of the Bar			
Characteristic	Symbol	Measure Unit	Value
Bar length	$l_{c}$	(mm)	8000
Outer diameter of the circular cross-section	$l_{c}$	(mm)	100
Inner diameter of the circular cross-section	d	(mm)	80
**B. Properties for S355 Steel** [19]			
Young’s modulus	*E*	(MPa)	$2.1\cdot 10^{5}$
Normal stress at yielding	$f_{y}$	(MPa)	355

**Table 2:** Values of the ratios $k_{1}$ and $k_{2}$ considered for the analyzed cases with the FEM for bars with pin connections at one end and simple supports at the other end.

No.	Code of Case Analyzed	Type of Column	Ratio *k*_1_ * between the Second Moment of Inertia of Portions	Ratio *k*_2_ * between the Lengths of Bar Portions
1	CONST_1I	Column with constant cross-section whose moment of inertia was I	1	0.0
2	STEPWISE405	Column with stepwise variable cross-section	4	0.5
3	STEPWISE410		4	1.0
4	STEPWISE420		4	2.0
5	STEPWISE430		4	3.0
6	CONST_4I	Column with constant cross-section whose moment of inertia was 4*I*	4	0.0

* Ratios *k*_1_ and *k*_2_ are shown in Figure 1a.

**Table 3:** Comparison between the critical buckling force $P_{cr}$ obtained by the FEM with the one obtained with the analytical model for different cases of bars involved in this research.

Code of Case Analyzed *	$\mathrm{Critical Force}\;P_{cr}$ of Stability Loss (kN)		Error (%)
	FEM	Analytical Model	
CONST_1I	93.8546	93.807	0.05
STEPWISE405	159.2584	165.620	3.84
STEPWISE410	230.5490	230.430	0.05
STEPWISE420	321.9348	312.270	3.10
STEPWISE430	346.3275	346.150	0.05
CONST_4I	375.4182	375.228	0.05

* Details about bars of each case are given in Table 2.

## Data Availability

Not applicable.
